# Ten simple rules for delivering live distance training in bioinformatics across the globe using webinars

**DOI:** 10.1371/journal.pcbi.1006419

**Published:** 2018-11-15

**Authors:** Denise Carvalho-Silva, Leyla Garcia, Sarah L. Morgan, Cath Brooksbank, Ian Dunham

**Affiliations:** 1 European Molecular Biology Laboratory, European Bioinformatics Institute (EMBL-EBI), Wellcome Genome Campus, Hinxton, Cambridgeshire, United Kingdom; 2 Open Targets, Wellcome Genome Campus, Hinxton, Cambridgeshire, United Kingdom; Dassault Systemes BIOVIA, UNITED STATES

## Introduction

Bioinformatics learning opportunities are now easily available face to face [1] or online [2]. As a rule of thumb, the former can (and will) trump the latter for its level of interactivity and engagement [3]. Most, if not all, students appreciate having the trainer (and classmates) available and close by. Their questions will get answered on the spot, on a case-by-case basis, with a personal touch. If the students happen to be in Europe (e.g., [4,5]) or North America (e.g., [6, 7]), they are in luck: there is no shortage of opportunities for such engaging encounters. Funding is often available for these students to attend face-to-face training. However, other parts of the globe tend to get neglected when it comes to live (and lively) face-to-face scientific training. Although capacity-strengthening initiatives, such as the Pan African Bioinformatics Network for Human Heredity and Health in Africa (H3ABioNet) Initiative [8], CABANA [9], Asia Pacific BioInformatics Network (APBioNet) [10], attempt to address this inequality, especially in low- and middle-income countries, scalability will always be an issue for face-to-face training. Online courses [11–13], however, allow training at scale, regardless of the trainees’ location. Funding for travel is no longer a hurdle: the only requirement is access to a computer (perhaps a smart phone or tablet) and an internet connection. The course is taken in the comfort and convenience of the trainee’s home, office, a library, or perhaps a coffee place with free Wi-Fi. However, on-demand access can be offset by lack of interactivity. Although online training portals often have chat rooms or other means of interacting with fellow learners or the course provider, discussions initiated this way often have a lag time.

Web-based seminars (webinars) offer the best of both worlds: they are run online and therefore at no (or little) cost for trainees, they can be scheduled at a convenient time for the target audience, the geographic distance between the trainer and the trainee is no longer an issue, and they allow for interaction between trainer and trainees at the moment of delivery. Webinars are short and straight to the point; the duration is usually no longer than 60 minutes. Questions are encouraged. Quick polls can be launched at any time for further interaction and getting to know the audience. Hands-on exercises can be provided, and follow-up webinars can be arranged for further discussions.

How can you achieve a stress-free and successful live streaming of bioinformatics training, which is interactive and available to everyone everywhere? Here are 10 simple rules that we have developed over the past five years of organising and delivering webinars [14]. Although our 10 simple rules are designed to deliver training on bioinformatics resources and projects, they can be easily applied to other domains. Due to the low cost, short duration, and flexible, potentially global access, webinars can be used to train and/or promote a variety of themes in bioinformatics, computational biology, and computer science. Webinars will shorten the cycle time of your training and give you leeway to broaden your arsenal of content. You will be able to cover examples on protists, bacteria, plants—typically of great interest in low- and middle-income countries [15]—and have time to explore new trends in the application of machine learning, artificial intelligence, and blockchain in life sciences. Despite the possibilities of different contents, please be aware this article is not about selecting a training topic but rather on delivering training using webinars.

## Rule 1: Choose your webinar software wisely

There is no shortage of “best webinar software products” recommendations out there. You have options to suit every pocket, operating system (e.g., Apple, PC, Linux), and internet browser (e.g., Internet Explorer, Google Chrome, Firefox) and to accommodate small or big audiences. Free products have the disadvantage of limiting your audience size: there are a limited number of “seats” for attendees, typically 30 in a single webinar session. If you want to reach out to as many people as possible, free software is therefore unlikely to be an option. You should allocate some money in your budget to account for that. See Table 1 for our top five software programmes, with the minimum requirement of must-have features, other key specifications, and the URLs to learn more.

**Table 1:** Top five webinar software programmes.

Webinar/training software	Software installation	Maximum capacity	Storage space	Operating systems compatibility
GoToTraining	Yes, local download	200	2 GB	PC, Mac
GoToWebinar	Yes, local download	1,000	Unlimited	PC, Mac
Cisco WebEx training	No, directly through a web browser	100	1 GB	PC, Mac, Linux
Zoom video webinars	No, directly through a web browser	10,000^a^	1 GB	PC, Mac, Linux
Myownconference	No, directly through a web browser	2,000	Unlimited	PC, Mac

These five software programmes all share a minimum requirement of features, namely polls, evaluation, chat, screen sharing, recording, telephone integration, mobile device (with application), and custom registration (i.e., branding, logo).

^a^Maximum capacity can be unlimited if broadcast via Facebook Live or YouTube.

You must also take into account other perks of the software you purchase. These are some useful features of your platform-to-be:

- Can you track registration and attendance?
- Can you share the screen during the webinar?
- Can you launch polls in real time?
- Can you collect feedback after the session?
- Can you obtain detailed analytics?
- Can you text chat with the audience?
- Can you record the session?
- Can you assess the attention level of the audience?
- Can you follow up with emails?
- Can you share the webinar materials (slides, exercises)?
- Can you integrate the software with other tools (calendars, social media channels)?
- Can you change the control of the screen in real time to a presenter other than yourself?
- Can you copy (or export) the results of evaluation surveys into other tools?
- Can you copy evaluation surveys from other tools into the webinar software?
- Can you easily get hold of the support team in the event of a “webinar catastrophe?”
- Can the participants dial in using a telephone line in the event of poor Wi-Fi connection?
- Can the participants join webinars on the go with a mobile device?

If you can answer “yes” to most or all of the questions above, consider purchasing a license for that software. If not many questions get “yes,” think about which of the items above are a must for you. Which ones can you live without? Go out there, do your research, get a free trial, carry out a dry run (or several), and move on to our Rule 2.

## Rule 2: Pilot it with celebrities and friends

Now that you have the webinar software of your choice, you should pilot it to get familiar with the infrastructure. You will need a topic and an audience for this. The right topic is the one that you know a lot about (and/or work with) and there is a need for training on. The audience is a group of people who you know and who can give positive and constructive feedback.

At the European Molecular Biology Laboratory, European Bioinformatics Institute (EMBL-EBI), we chose to pilot our webinar software with well-known and widely used bioinformatics resources (hence “celebrities”), namely PDBe [16] and Ensembl [17]. Moreover, both resources have had global user communities and well-established face-to-face training [18,19] and online presence [20,21]. Our friendly audience (hence “friends”) were members of the Industry programme [22], have had a close relationship with our training activities, were generous with their time, and were keen on exploring alternative means of training.

If you are a computational biologist or bioinformatician, you can pilot your webinar software on topics such as next-generation sequencing data analysis and data visualisation. The audience of friends could be bioinformaticians or (computational) biologists you have worked with, or colleagues in the bioinformatics facility (institution) you work at. Because many research-performing institutes—from universities to companies—have experienced training and development teams, you can engage with them and bring them into the discussion from the beginning.

Make sure you run this pilot within the free trial period of the webinar software. Once the pilot is done, check how well your software performed against the list of perks from Rule 1. If it did not perform well enough for the license you have to pay, try a different software. If you need to run several pilots until you find the software that works for you, or if you are in shortage of friends who are willing to be your audience, you can pilot your webinar with just one person, e.g., a work colleague or facilitator—see Rule 6, or go solo altogether.

## Rule 3: Get a host on board

If face-to-face workshops are already part of your training portfolio, the chances are you have a list of people who have hosted your workshops in the past. Get in touch with the previous hosts and invite them to host a webinar, as they will help you to find the right audience (see Rule 4). Having a host on board will take the pressure off of you so you can focus on the important things such as crafting the syllabus, writing the abstract of the training, and creating the webinar registration link (from the webinar software of your choice—see Rule 1). Remind them of the advantages of hosting a webinar (e.g., save money, time, and effort). They just need to forward your webinar registration link to their contacts. Once you become more confident with online training and get the gist of it, you may want to ditch the need for a host. And jump straight on to Rule 4.

If you have never delivered face-to-face training before, you will not have a list of hosts. If that is the case, consider getting in touch with your network of work colleagues (past and/or present) and tell them about your training using webinars. They may be able to find a suitable audience for you. If you want to deliver your webinar to a much broader audience, share the registration link via mailing lists and social media (e.g., Twitter, LinkedIn, Facebook, Medium, blog).

## Rule 4: Find your audience

Before advertising your webinar, save a few spaces for members of your team (possible supporters at the Q&A session—see Rule 7), in case your webinar is a sell-out (i.e., all places get filled up). To cast your net as wide as possible and increase the number of registrants, advertise your webinar on social media [23], virtual conferences [24], and promotional events where online presence is encouraged such as festivals and open days, mailing lists (BioStars, Reddit), newsletters, and relevant journals. You can also go back to your friends (see Rule 2) and ask them to share the webinar registration link.

Keep checking the usernames, email domain, and number of registrants if possible. Watch out for likely bots by looking through their registration. If names and/or usernames contain random characters (alphabetical or numerical) only, it is likely to be fake registrants. You do not need to do anything with this at that moment. Carry on advertising the webinar widely, but do check the bot suspects on the day, especially if they do not turn up (see Rule 6). You might not be able to do anything against bots, but you will keep this in mind to get a more realistic expected number for genuine attendees.

## Rule 5: Prepare your content: Less is more

You love what you do—the bioinformatics resource or project you work on—so it is natural that you will try to cram in a lot of information you think is relevant. Do not! The audience will likely not remember every tiny detail you include in your webinar. And as a distance learning activity, it is less straightforward for assessing whether you have lost them or they are still with you with full attention (see Rule 6).

Start small by only giving an overview and some highlights of your topic so that you do not end up with a large number of slides to present and having to rush through them. You can offer follow-up webinars to cover additional content and train your audience on the finer details of your topic (this can be part of a series of webinars). Do not overcrowd your slides with detail; have them as visually compelling as you can. Consider adding live demos or play a recording (.mov, .mp4, .gif) showing some functionalities of your resource, either in a graphic or programmatic way.

It is good practice to build a couple of polls into your webinar to hold your audience's attention—these can be general questions like asking them which country they are listening in from, or they could be more specific to your topic, such as whether they have used the method, service, and/or resource that you are about to describe.

Include a slide on the logistics of the live webinar, such as:

- Webinar attendees will be muted.
- Materials (slides, exercises) will be available for download.
- Polls will be launched during the webinar.
- Attendees are encouraged to ask questions, which should be addressed to everyone.
- Questions will be answered at the end (via chat box or otherwise). Note: if you have a colleague with you, you could agree with them to answer any questions while you deliver the webinar. If that is the case, introduce anyone else who is assisting you at the session.
- The session will be recorded and shared.

At this stage, you also need to think about your post webinar survey. We will tackle this in more detail in Rule 9 but for now, just consider adding a “survey” holding slide at the end of your presentation; this will ensure that you do not forget to mention the survey at the end of your talk. Finally, practice your talk, make sure you will not run over, and check whether it flows smoothly throughout.

## Rule 6: Lights, camera, action

The big day has finally arrived. You may have a full house or just a handful of registrants. Both are great. Even if not many have registered, you will be recording your session (see Rule 8), so the video will be available for anyone to watch it. It is very unusual to get a full turnout—more typical is that 40% to 60% of registrants turn up. A few could be bots (see Rule 4 on basic tips to spot them), but most are likely to be people who were genuinely interested in attending but have failed to do so because something more pressing came up. Do not feel let down. Monitor the level of attendance in future webinars and try to establish whether there is a pattern. If you are suffering from frequent low attendance, consider the following: was the timing wrong? Did your webinar overlap with other events on the same topic? If you are webcasting to a single host, did it clash with a regular seminar or meeting taking place at the host organization? You could consider sending them a message saying “We were sorry you missed our live webinar; here is the URL link to the recording and materials. Do let us know if you have any further questions or if we can do anything to make our live webinars more convenient for you.”

You should now get your laptop ready for the live session. Make sure you close down email clients, Slack, Skype chats, and so on. You do not want notifications popping up at the corner of your computer screen or beep sounds during your training session. Do not deliver your webinar from your regular desk. Book a room instead to ensure you are in a quiet place and will not be interrupted during the webinar. Have your laptop plugged in and use a wired internet connection. If neither is possible, make sure your laptop is fully charged and that the Wi-Fi internet connection is working. Remember some of the basics for ergonomics. Bring your keyboard and mouse, rather than using the laptop’s unfriendly counterparts. Get the screen at eye level so that your head and neck are at a comfortable position. Having the right eye level means that attendees will be looking into your eyes rather than eyelids or forehead if you choose to have your built-in camera on. Feel free to experiment with this setting. There is no “one size fits all.” You can have someone with you facilitating the webinar (e.g., opening and closing polls—see Rule 7—and answering the questions that get asked while you deliver the webinar). Having a facilitator especially when you first start delivering webinars will let you focus on the content rather than worrying on how to fix any possible technical issues that may arise, thus reducing the risks of things going awry. We recommend that the facilitator be in the same room as you. Once you have delivered more webinars and mastered some of the rules above, you can have facilitators remotely, or you can go completely solo, which is possible once you become more confident with webinars.

Some software has a beep sound to notify you when people join or leave the training session. Disable it. It is annoying for the attendees and distracting for you. Focus on delivering the session rather than on the sounds of attendees joining in later or leaving earlier. Let your audience know this is your first live, training webinar.

## Rule 7: Be engaging

A golden rule when it comes to training is to know your audience and engage with them. One of the ways you can do so during your webinar is to make use of polls—the webinar software you purchase should offer this functionality (see Rule 1). Polls are short and simple questions that you build before you go live. You can ask, for example, which species your audience works on, which computer language(s) they code in, whether they have used your resource, which other similar resources they have used, and so on. Since you are new to webinar delivery, it is best to practice these beforehand.

Once you launch the poll live, watch the votes coming in and the overall percentage of votes, wait a minute or so, and then close the poll. Thank the audience for answering the question and share the results with them. The answers will help you pitch the level of your webinar even if your slides are already uploaded and shared with the audience. If it turns out, for instance, that the audience is mainly of users who already know the resource, you can skip the basics. Or if you learn that none of registrants is computer savvy, you can skim through the programmatic access. Just make sure you and your presentation are flexible enough so you adjust the content to your audience on the fly.

In addition to regular polls, you can also throw in direct questions and tell the audience to respond via chat. Besides allowing you to be more spontaneous, direct questions can be used for cases in which answers do not fit in the simple multiple choice format of polls. If you want to know where your audience is listening in from, throw that in rather than using a poll because the latter typically does not allow more than four of five different options.

Another way to engage with your audience is encouraging them to ask questions, during or after the webinar, and via the chat box or the speaker. It is entirely your choice. Experiment with the different formats, then choose which ones you are more comfortable with. Our choice is to take all questions at the end. The reasons are 2-fold. Firstly, you may be about to cover what an attendee has just asked. Secondly, you have only typically 30 to 60 minutes to cover your content, and questions during the training may leave you short of time. If you have a colleague (or facilitator), you can introduce her/him and say that they will be happy to answer the question via the chat box while you talk. This is an exception because it is not always possible to have a facilitator. In the absence of one, check the chat box at the end and answer the questions once your presentation is over. Whatever your choice, make these housekeeping rules clear in your logistics slide (see Rule 5).

We also ask the audience to address the question to “everyone,” preventing another attendee asking the same question. Besides, having the questions available to everyone allows for attendees to chip in (if they wish) or engage with the discussion, which adds an extra flavour to the engagement mix. Another advantage of having a written Q&A is that you can keep them as a record of the pain points and/or feedback.

If you choose to do the Q&A verbally, do so at the end of your webinar (for the same reasons outlined above) and unmute all the participants. Be aware of likely echoing once the microphones are no longer muted, especially if the participants happen to be in the same room. Also take into consideration that speaking may put some attendees off from asking questions, especially if the language of the webinar is different from their mother tongue. They will probably prefer writing the question down rather than speaking it out loud.

## Rule 8: Record the session and share it

Webinars are “for life, not just for Christmas”; i.e., they are not just for that single time of broadcast. Record your session to make it available to those registrants who did not make it to the live webinar. Share the recording more widely (perhaps by posting to a service such as YouTube or Vimeo) and make it available to a much broader audience. Do not make the mistake of delivering the webinar and at the end realising you did not hit the “record” button. Press the button for both screen and voice even before the session starts. If you still forget to hit the record button, or if there are issues with the recording, there is nothing to stop you from delivering the same webinar again, this time with no audience, and making a clean recording available. Once the recording is done, go through it before sharing it. You may need to edit it (but do not spend too much time doing so) to remove long pauses, the start and the end of the recording, and the Q&A session. Once you have the final version, make the recording available from a website, e.g., the tutorial page of your own resource [25] or from a video-sharing website [26–29]. Video-sharing websites often have a social media element that can pull in a new audience previously unaware of your bioinformatics resource, so it is worth considering both approaches. Whichever approach you take, use your social media network to disseminate your new video. Regardless of the number of attendees in the live webinar, once the recording is shared, it will function as an online resource, which over time will reach a much wider audience.

## Rule 9: Get feedback and act on it

Hooray! You have delivered your webinar, the recording is online, and you are ready to move on to deliver more webinars. Before moving to the next, you need to assess how the first one went. Seek feedback on the style, the content, and/or the technical aspects of your training session. Evaluation surveys are the first channel of feedback that comes to mind. You can choose to send these as soon as the webinar is over, an hour later, or perhaps the day after. The sooner you send this, the higher the chances that the attendees will fill it out. It helps to know how to write surveys that work [30,31]. Our surveys are short (four to five questions) and have a combination of open questions and multiple choice (three to four options per question). We ask attendees to rate the session (from 1 [poor] to 5 [best]; be aware that some respondents may get confused and think 1 is best and 5 is poor). Precourse surveys are less likely to be representative of the audience and do not work for webinars because attendance rates are typically considerably lower than the number of registrants.

The Q&A at the end of the webinar (see Rule 7) is also a chance for the attendees to “voice” their final comments, which tend to be positive feedback. They can vary from “thanks,” “enjoyed a lot this webinar,” “I learned quite a lot” to “great presentation,” “perfect introduction to this resource,” “it looks like a really useful tool (amazing job)!” Make a note of them. Once your webinar is published (see Rule 8), monitor future engagement with it through comments and thumbs up or down on video-sharing websites.

Be objective when acting on the feedback, especially negative feedback. Some issues may be a matter of style (e.g., whether to show a thumbnail video window or not). Others can help you not to make the same mistake again or decrease its likelihood (e.g., once we launched a poll but forgot to close it, and the presentation froze). Look at the feedback as a standard normal curve: one may find it exceptional, another may find it mediocre; see what the majority says, and base your actions primarily on that. If you do not agree with the feedback, it may help to share it with colleagues for a second opinion. You can modify your materials (or delivery) accordingly. Besides feedback on content and style, participants may also highlight technical hiccups. Your software of choice may not be the best for their operating system or choice of internet browser. If that is the case, you can build recommendations into your registration confirmation email or housekeeping slides for future webinars. If you have had your camera on during the webinar, the feedback can be on the settings (e.g., light in the room being too bright or too dark, the wall behind the speaker too distracting, poor quality of the sound, and/or network speed). Take note of those issues and advise the forthcoming participants of some of these, so you (and they) do not run into similar issues. Overall, you will be able to act on the feedback received and adjust the content and level of detail if necessary in future webinars.

## Rule 10: It is not the end

Once webinars become part of your routine, you may tend to believe that this is it. You have nailed it. It is the end. Far from it. It is actually the beginning. The webinar can be a flavour of what is to come. Now that your audience has had a taste of the training that you can deliver, they may invite you to deliver face-to-face training. Or you might find that webinars are an effective means of providing updates on a bioinformatics methods or resources.

Things you may want to consider for future webinar training sessions are as follows:

- How often will repeats be required?
- Will you be the speaker again, or will you invite others?
- How can you expand the pool of speakers?
- Where will you get your audience from?
- What should you do if my audience wants to use a webinar software that is different than the one of your choice?

Be open and flexible: webinars may not suit everyone or every topic. There may be distinct advantages to meeting face to face with an audience or developing a more “static” online tutorial to answer frequently asked questions. It is not a matter of which one is best; face-to-face training, tutorial-based e-learning, and webinars all have pros and cons. We hope that these 10 simple rules will be tried, tested, and adjusted as you see fit. Let us know how it goes.

## Conclusion

Webinars are a powerful and engaging means of training and dissemination [32] that can reach global audiences and therefore help us to address inequality and imbalance of teaching bioinformatics or other subjects. How can you deliver a stress-free webinar that can be available to everyone everywhere? Our 10 simple rules will decrease the activation energy of delivering webinars: you will feel better equipped to adopt this alternative method of training and take your subject of expertise to places where face-to-face training is not available. We hope that our experience can inspire you into this brave new world of live distance training. You will then be able to add your own rules, especially if you decide to use webinars in contexts other than training only.
